# Phenotypic heterogeneity in Pseudomonas aeruginosa virulence revealed through passive exposure in zebrafish (Danio rerio) and nematodes (Caenorhabditis elegans)

**DOI:** 10.1099/acmi.0.000748.v4

**Published:** 2026-10-20

**Authors:** Daniella Azulai, Anielka Espinosa, Maracela Talamantes, Heather L. Bennett, Gabriel G. Perron

**Affiliations:** 1 Department of Biology, Reem-Kayden Center for Science and Computation, Bard College, Annandale-On-Hudson, NY 1250, USA; 2 Department of Biology, Trinity College, Hartford, CT, 06106, USA; 3 Center for Environmental Sciences and Humanities, Bard College, Annandale-On-Hudson, NY, 12504, USA

**Keywords:** *C. elegans*, host–pathogen interactions, model system, nematode, opportunistic infection, *Pseudomonas aeruginosa*, virulence, zebrafish

## Abstract

The rapid emergence of diseases and parasites in aquatic wildlife requires improved methodologies to identify and characterize new and future pathogens. While microinjection of pathogens directly into a model organism, such as zebrafish, enables the exploration of infection and immune response in the host, such methodology focuses primarily on identifying causative agents in events of aquatic wildlife mortality due to acute infection. Here, we present an updated protocol of infection by static immersion in larval zebrafish to investigate the possible effect of prolonged environmental exposure to an opportunistic pathogen. By controlling microbial growth and monitoring mortality over 72 h, we show that static immersion can detect minute differences in virulence profiles between and within different strains of *Pseudomonas aeruginosa*, an important opportunistic pathogen of animals and humans. We then conducted two sets of passive exposure virulence assays in *Caenorhabditis elegans*, an alternative model. We demonstrated the virulence phenotype, while showing slight differences between experimental models, and showed similar trends. We believe that passive exposure thus offers a practical host–pathogen model that simulates opportunistic infection occurring in the environment and enables the detection of minute changes in virulence between and within bacterial strains.

## Data Summary

The raw survival data files for each trial described in this study, as well as the code used for generating and analysing the survival curves, are available at the Open Science Framework (DOI: https://doi.org/10.17605/OSF.IO/MEYDJ).

## Introduction

The rapid emergence of diseases and parasites affecting wildlife organisms is of increasing concern [1]. While parasites can be a natural part of healthy ecosystems [2], environmental disturbance and climate change have increased parasitic load in many environments [3, 4]. Such outbreaks of infectious diseases are especially problematic in organisms for which environmental changes can directly impact physiological responses, such as fish [5, 6] and insects [7, 8]. In addition to hindering conservation efforts [9], the increased burden of parasites in wildlife increases the risk of introducing novel pathogens in human populations [10].

Several factors contribute to the emergence of novel pathogens in wildlife. Environmental stressors such as antimicrobials can impair host immunity [11, 12], creating ecological opportunities for normally benign microbes to become pathogenic. Similarly, genotypic and phenotypic diversification in bacterial populations can result in the evolution of novel virulence traits [13]. These dynamics are particularly prominent under source-sink population structures [14], where increased environmental bacterial density raises the mutation supply rate [15, 16]. As a result, investigating genotypic and phenotypic diversity in environmental bacterial reservoirs is essential for predicting pathogen emergence [17, 18]. Yet, the study of natural populations of bacteria remains sparse [5, 19].

*Pseudomonas aeruginosa* is a striking example of an opportunistic pathogenic bacterium that can successfully invade a range of hosts and new hosts [20]. The aquatic and soil bacterium can infect various organisms such as plants, nematodes, fruit flies, wax moths, zebrafish and mammals under certain conditions [21]. The bacterium is also associated with high rates of morbidity and mortality in intensive care unit patients. Indeed, even within a single host like humans, *P. aeruginosa* can invade various tissues and sites [22–24]. *P. aeruginosa*’s pathogenicity is due to various virulence factors and antibiotic resistance determinants, providing the bacterium with metabolic flexibility and great adaptability [25, 26].

To better understand how phenotypic heterogeneity influences *P. aeruginosa* virulence, model systems capable of capturing a wide range of host responses are needed. The zebrafish (*Danio rerio*) is a well-established vertebrate model with an immune system that shares considerable similarities with mammals [27, 28]. While microinjection of pathogens is the preferred methodology in this host [29], passive exposure via static immersion is more characteristic of a large number of fish pathogens, including *P. aeruginosa*, transmitted via contact with the eyes, gills, skin or even the gut following ingestion [30, 31]. Interestingly, previous work showed that *P. aeruginosa* strain PAO1, a common lab strain, did not yield mortality in larval zebrafish following a short exposure (i.e. 5 h) but triggered genes associated with a stress response [32].

Another widely used model for studying host–pathogen interactions is *Caenorhabditis elegans*, a small nematode with a shorter developmental frame that is also susceptible to human pathogens [33]. Indeed, the nematode enabled the identification of various virulence-related genes in *P. aeruginosa* via transcriptional changes in *C. elegans* exposed to the bacterium [34–36]. Interestingly, *C. elegans* predominantly feeds on bacterial cells, thus providing a different mode of entry for pathogenic bacteria.

Here, we examine virulence heterogeneity in five *P. aeruginosa* strains using both zebrafish embryos and *C. elegans* as model hosts. By standardizing bacterial exposure and monitoring host mortality over time, we demonstrate that passive exposure not only confirms virulence in both models but also reveals significant variation in virulence between and within bacterial strains across hosts.

## Methods

### *P. aeruginosa* strains

We selected five *P. aeruginosa* strains from a more extensive collection at Bard College (Table 1). Clinical *P. aeruginosa* strains used in this study were chosen to reflect the diversity of phenotypes observed in the bacterium, including different infection sites, countries and dates of isolation. More information for each strain is available elsewhere [21, 24]. This panel was selected to capture a broad range of virulence-associated traits among clinically relevant strains: PAO1 is a widely used, laboratory-adapted strain, whereas the remaining isolates are associated with differing antibiotic resistance profiles and with acute versus chronic infection presentations. We did not include a non-pathogenic environmental isolate in this panel; such a comparison would help establish a lower bound of virulence in these models and would represent an important direction for future work.

**Table 1.** List of *P. aeruginosa* strains

Strain name	Country	Date isolated	Source
A10	France	1882–1918	Wound
AGO4092	Greece	1994	Urine
ESP06B	Belgium	1993	Clinical
Lw1047	Congo	2001	Blood
PA01	Australia	1955	Wound

To control for potential differences in virulence phenotype due to changes in growth between the strains of *P. aeruginosa*, we started each passive exposure assay with the same concentration of bacteria. While *P. aeruginosa* strains were grown on Luria-Bertani (LB) agar plates in *C. elegans* assays, the bacterium was maintained in 1× E3 media (i.e. 5 mM NaCl, 0.17 mM KCl, 0.33 mM CaCl_2_ and 0.33 mM MgSO_4_; pH 7.2) in zebrafish static immersion assays. For the latter, we measured bacterial growth by inoculating the bacterium at three concentrations (e.g. 10^3^, 10^5^ and 10^9^ c.f.u. ml^−1^) in E3 medium. The bacteria were incubated at 28 °C, and growth was monitored at 12 and 24 h using serial dilution (Fig. S1A, available in the online Supplementary Material). Finally, to test whether *P. aeruginosa* subsisted in E3 media due to leftover LB nutrients following overnight culture, we compared the growth of *P. aeruginosa* starved in M9 media for 8 h to that of *P. aeruginosa* washed in sterile 1× E3 media [37]. In addition, both treatments were grown in either 1× E3 media or 1× E3 media containing 1% LB to test the effect of added nutrients on bacterial growth directly (Fig. S1B).

### Zebrafish husbandry and static immersion virulence assay

WT Tu zebrafish were maintained at the Bard College Biology Department’s zebrafish facility. The fish were kept on a 14/10 h light/dark cycle in standard recirculating rack water maintained at 28 °C with pH ranging from 7.0 to 7.4. Fish mating was performed in standard mating reservoirs. Upon release, embryos were cleaned by immersion in a 0.5% bleach solution for 5 min, followed by deionized water for 5 min; then, the former steps were repeated. After washing, embryos were transferred to 100 mm^2^ Petri dishes in densities of 30–50 embryos and suspended in sterile 1× E3 media. Seventy-five per cent of the media was changed daily while embryos remained in the Petri dish.

Larval zebrafish were then used to conduct a static immersion virulence assay adapted from van Soest *et al*. [32]. While the latter exposed the larvae to a high concentration (10^9^ c.f.u. ml^−1^) of *P. aeruginosa* for 5 h, we exposed the animals to a lower concentration (10^5^ c.f.u. ml^−1^) of *P. aeruginosa* for 72 h. *P. aeruginosa* populations were prepared from an overnight culture grown in LB at 37 °C, centrifuged and resuspended in filter-sterilized 1× E3 media at the desired concentration. We placed one 3-day-post-fertilization (dpf) larva in 1 well of a 24-well plate, with each well containing 2 ml of the bacterial mixture. All assays were conducted in a well-oxygenated closed environment at 28 °C for 72 h. Fish mortality was then monitored every 12 h by observing larval movement and confirmed by touching immobile larvae with a sterile pipette tip. As a control, we also monitored the mortality of 24 larvae growing in 2 ml of sterile 1× E3 media, and each assay was conducted twice with independent broods. All methods described herein concur with humane animal testing standards and were approved by the Bard Institutional Animal Care and Use Committee (IACUC; approval ID ‘Perron 2018’.

### Maintenance of *C. elegans* and passive exposure virulence assay

WT, or N2, worms were obtained from the *Caenorhabditis* Genetics Center. Animals were cultured and maintained with standard methods [38]. *C. elegans* were grown on nematode growth medium (NGM) plates seeded with the *Escherichia coli* strain, OP50, at 20 °C. To synchronize worms by age, animals were bleached using a standard protocol [39], where gravid adults were dissolved in a buffer solution containing 4% sodium hypochlorite and 5 M sodium hydroxide. The *C. elegans* embryos that survived were then washed with M9 buffer twice and allowed to hatch on NGM plates again seeded with OP50 at 20 °C. Two days later, L4 worms were washed three times with M9 buffer before being used in subsequent assays.

For our passive exposure virulence assay, we used the slow-killing (SK) plates described elsewhere [33, 35]. Approximately 30 triple-washed and synchronized L4 worms were plated onto clean SK plates seeded with the standardized liquid culture of bacteria. Worms were kept at 25 °C, and every 2 days, living worms were transferred onto freshly seeded SK plates such that they were repeatedly exposed to the same bacterial strain. We monitored mortality by prodding immobile *C. elegans* every 24 h for 7 days following the first exposure to the bacteria. If any *C. elegans* were unaccounted for, they were recorded as missing unless found within the next day.

### Microscopy

We used microscopy to assess the possible presence of *Paramecium* in the E3 medium, a common contaminant in zebrafish experimental environments. Microscope images were obtained using a Nikon Eclipse E600 at 4× magnification. Fishes were placed onto a glass microscope slide suspended within a drop of E3 media using a sterile pipette. Fluorescence images were obtained using the Nikon Eclipse E600 at 4× magnification, equipped with the UV-1A filter cube attachment. Both brightfield and fluorescence images were acquired with a Jenoptik ProgRes MF Cool CCD camera; fluorescence excitation was provided by an Excelitas X-Cite mini+ LED illuminator (UV-1A cube: excitation 360–370 nm, dichroic mirror 400 nm and barrier filter 400 nm). Scale bars in Fig. S2 (500 µm) were calculated from the camera’s sensor pixel size (6.45 µm) combined with the objective magnification (4×) and the camera manufacturer’s standard 0.63× C-mount relay lens (2.56 µm/pixel); a stage micrometre calibration was not performed for these images. While we sometimes observed a suspected *Paramecium* bloom 24 h after larval death (Fig. S2A), we did not detect any substantial *Paramecium* populations during the survival stage of the experiment.

### Statistical analyses

To investigate the effect of growth conditions of *P. aeruginosa* growing in 1× E3 media, we used an analysis of variance to compare growth after 24 h, measured as c.f.u. ml^−1^, between populations initially inoculated with 10^5^ and 10^8^ c.f.u. ml^−1^. We then compared bacterial growth after 24 h in bacterial population inoculated with 10^5^ c.f.u. previously grown in LB or starved in M9 salt for 8 h before inoculation.

To test for heterogeneity in bacterial virulence, we compared animal survival for both zebrafish and *C. elegans* using the Kaplan–Meier estimate of survival. The latter was implemented in the *Survival* package of *R* [40], where each death is weighted using a log-rank test, and missing animals are given a value of 0 when they are not found. Throughout the Results, survival comparisons are reported as test statistic (*χ*²), the *P*-value adjusted for multiple comparisons (adj-*P*) and the corresponding figure panel. Finally, to test for a possible batch effect, we compared the two independent trials conducted for each model system using the Mann–Whitney test, a non-parametric test comparing the sum rank of two groups, and we compared the two independent assays performed for each bacterial strain–model system pair. All statistical analyses and model assumptions were tested in *R* 3.5.2 [41]. Graphs were plotted using *ggplot2* [42].

## Results

### Virulence in zebrafish

To establish a standard protocol to study bacterial virulence in an immersion assay, we first investigated the growth conditions of *P. aeruginosa* in the test medium. To do so, we monitored the growth of replicate populations of the bacterium inoculated at two different concentrations (i.e. 10^5^ and 10^8^) over 24 h. While the inoculation size had a significant effect on population growth (*F*_(1,4)_=31.85; *P*=0.005), we observed that all the populations increased in size, reaching on average 6.42 x 10^8^ and 1.67 x 10^9^ c.f.u. ml^−1^, respectively (Fig. S1A). Crucially, we also established that the bacterium’s growth was not due to residual nutrients stored in the bacterial cells or the inoculum due to prior growth in a nutrient-rich medium (*F*_(3,8)_=0.0092; *P*=0.99). These results suggest that *P. aeruginosa* can sustain a stable population in E3 media, providing an opportunity for virulence development. Based on our observation, we established that optimal static immersion conditions would be inoculation with 10^5^, thus allowing for stable bacterial populations in the medium.

We then tested whether long-term passive exposure to bacteria could result in mortality in larval zebrafish. To do so, we investigated the survival of zebrafish larvae exposed to five *P. aeruginosa* strains chosen to reflect the phenotypic diversity in the taxa. For each strain, we exposed 24 independent larval zebrafish randomly selected from a unique brood to identical bacterial growth conditions via static immersion in 2 ml microcosms. We then tracked larval mortality every 12 h. Overall, we found that all five *P. aeruginosa* strains induced increased larval mortality compared to a single mortality event in the control fish population (*χ*²=99; *P*<0.0001; Fig. 1a–e).

**Fig. 1.**
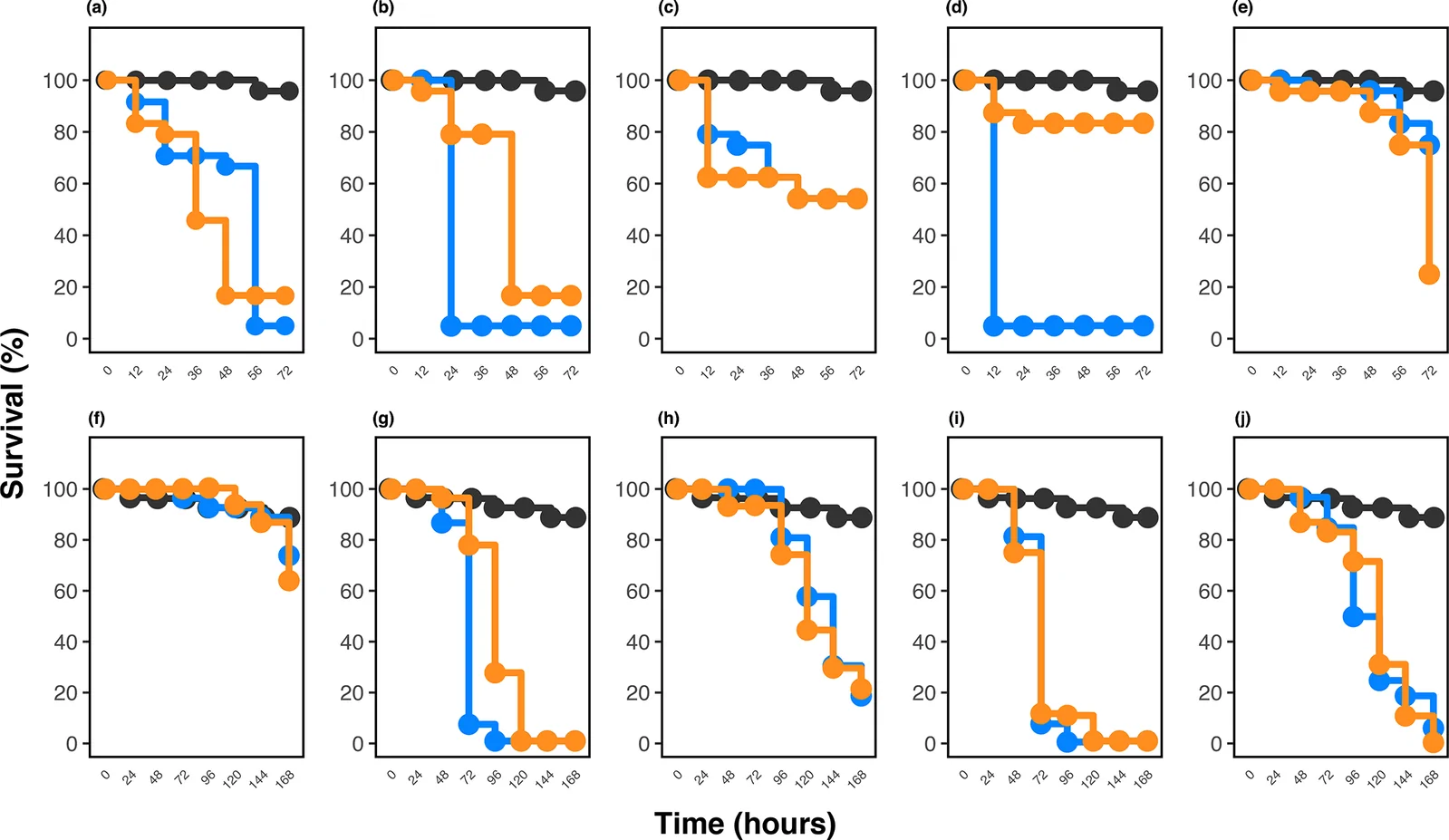
Survival curves of larval zebrafish and L4 *C. elegans* exposed to different *P. aeruginosa* strains. For zebrafish larvae, survival was computed every 12 h from populations of 24 zebrafish larvae exposed to *P. aeruginosa* strains (a) A10, (**b**) AGO4092, (**c**) ESP06B, (**d**) Lw1047 and (e) PA01, shown in orange (trial 1) and in blue (trial 2) and compared to a control population of 24 fishes not exposed to the bacterium (shown in grey). For *C. elegans*, survival of 30 *C. elegans* exposed to *P. aeruginosa* strains (f) A10, (**g**) AGO4092, (**h**) ESP06B, (**i**) Lw1047 and (j) PA01, shown in blue (trial 1) and orange (trial 2), and is compared to L4 worms exposed to OP50 (grey). For all tests, differences in survival were compared to control populations, and between each trial was tested using the Kaplan–Meier log-rank test..

Interestingly, we found that the *P. aeruginosa* strains induced mortality at varying rates. More specifically, we found that zebrafish exposed to strains A10 (*χ*^2^=49.3; adj-*P* <0.001; Fig. 1a) and AGO4092 (*χ*^2^=41.2; adj-*P* <0.001; Fig. 1b) displayed high mortality relatively early in the assay, with more than 80% larval zebrafish dying by 48 h post-exposure. Larvae exposed to strain PAO1 (*χ*^2^=19.5; adj*-P* <0.001; Fig. 1e) also suffered high mortality, but at a slower rate than the strains mentioned above, with more than 80% of larvae dying at 72 h post-exposure, correlating with the development of mouth opening in the zebrafish larvae at ~5 dpf. Fish exposed to strain ESP06B also showed lower mortality rates (*χ*^2^=13.1; adj-*P*=0.0015; Fig. 1c), but still significantly more than the control. Finally, fish exposed to strain Lw1047 did not show significant mortality compared to controls (*χ*^2^=0.9; adj-*P* >0.99; Fig. 1d).

While strains A10 and AGO4092 were not statistically different from each other (*χ*^2^=6.5; adj-*P*=0.1), they differed from all other strains (adj-*P*s <0.05). In addition to being statistically different from strains A10 and AGO4092, strain PAO1 was statistically different from strain Lw1047 (*χ*^2^=11.3; adj-*P* <0.001) but not strain ESP06B (*χ*^2^=0.2; adj-*P* >0.99). These results suggest that we observed three phenotype classes among the *P. aeruginosa* strains in zebrafish larvae: high (80% mortality), low (50% mortality) and absent.

To investigate the reliability of our results, we repeated the above static immersion assay with a second independent larval brood. Again, we exposed populations of 24 independent larval zebrafish randomly selected from a unique brood to identical bacterial growth conditions via static immersion in 2 ml microcosms for each strain. While we observed the same overall trends with strains A10 and AGO4092 showing high mortality and ESP06B showing the lowest larval mortality, survival curves were often significantly different for each strain between the two trials. To test whether survival patterns differed between the two independent trials for each strain, we compared trial 1 and trial 2 directly and we found a statistical difference in survival in fishes exposed to strains A10 (*χ*^2^=23.2; adj-*P* <0.001; Fig. 1a), AGO4092 (*χ*^2^=50.4; adj-*P* <0.001; Fig. 1b), Lw1047 (*χ*^2^=92.6; adj-*P* <0.001; Fig. 1d) and PAO1 (*χ*^2^=11.8; adj-*P* <0.001; Fig. 1e). This second trial confirmed the consistent presence of three virulence phenotypes but also demonstrated an important source of variability between trials.

### Virulence in nematodes

We then investigated whether *P. aeruginosa* would show a similar virulence profile in *C. elegans*. To do this, we fed independent populations of synchronized L4 nematodes on any of five different bacterial strains as described above. Control populations were fed on OP50, the standard laboratory bacterial diet. Survival for each nematode was scored every 24 h. Overall, we found that every *P. aeruginosa* strain, except for A10 (*χ*^2^=0.4; adj-*P* >0.99; Fig. 1f), increased mortality compared to *C. elegans* fed the control diet. Moreover, we found that *C. elegans* survival was significantly lower when exposed to strains AGO4092 (*χ*^2^=47; adj-*P*=0.002; Fig. 1g), ESP06B (*χ*²=23; adj-*P* <0.001; Fig. 1h), Lw1046 (*χ*^2^=30.4; adj-*P* <0.001; Fig. 1i) and PAO1 (*χ*^2^=30.3; adj-*P* <0.001; Fig. 1j). We also found that strains AGO4092 and Lw1047, while significantly different from each other (*χ^2^*=29.4; adj-*P* <0.001; Fig. 1g), had the highest mortality rate early in the assay, with only 6.67 and 0% of the exposed *C. elegans* surviving the first 72 h. Strains ESP06B and PA01, which did not differ significantly (*χ*^2^=4.8; adj-*P*=0.15), still led to 90 and 96% mortality, respectively, by the end of our experiment.

Again, to investigate the reliability of our results, we repeated the above assays with a second independent trial. Unlike our assays with the zebrafish model systems, we found that most bacterial strains tested showed similar virulence patterns over the two trials. Indeed, strains A10 (*χ*^2^=0.2; adj-*P* >0.99; Fig. 1f), ESP06B (*χ*^2^=0; adj-*P* >0.99; Fig. 1h), Lw1047 (*χ*^2^=0; adj-*P* >0.99; Fig. 1i) and PAO1 (*χ*^2^=0.2; adj-*P* >0.99; Fig. 1j) showed similar virulence profiles. Only strain AGO4092 showed a different virulence profile in the second trial, with a dip in survival happening after 72 h in the first trial and after 96 h in the second trial (*χ*^2^=25.8; adj-*P* <0.001; Fig. 1g). These results suggest that *C. elegans*, while allowing for variance in virulence between strains, might be a more stable model system between trials.

## Discussion

Here, we investigated the use of passive exposure in two animal models as a way to characterize virulence phenotypic variability in an important opportunistic pathogen. We show that passive exposure is indeed a reliable assay, exposing variability between and within strains of bacteria in both *D. rerio* and *C. elegans*. Specifically, we found that *P. aeruginosa* presents at least three broad virulence patterns that could be statistically differentiated. Indeed, the bacterium could induce complete mortality, partial mortality or no mortality depending on the host and trial. Overall, we found that virulence patterns were consistent between the trials and hosts, suggesting that there exists a strong genetic component to virulence in each bacterial strain.

Yet, we also found that some strains of *P. aeruginosa* harboured different virulence patterns between the two host models. For instance, strain A10 was highly virulent in zebrafish but showed little to no pathogenicity in nematodes, while strain PAO1 showed moderate virulence in both. These discrepancies highlight that host-specific factors, such as differences in innate immunity, microbiota composition or tissue tropism, may interact with bacterial virulence traits in complex ways. Our study thus highlights the need to consider genotypic and phenotypic diversity when investigating infectious diseases and that using more than one model could enable us to focus on different aspects of virulence in the bacteria.

The route of infection is a critical determinant of host–pathogen interactions, influencing both the establishment of infection and the ensuing immune response. *P. aeruginosa* is primarily an opportunistic pathogen that establishes infection through breaches in host barriers, such as damaged skin, the respiratory tract or medical devices, where it can adhere, form biofilms and evade host immunity. In contrast, passive exposure through ingestion is generally insufficient to establish infection in immunocompetent hosts, as the gastrointestinal tract provides substantial colonization resistance through the resident microbiota, mucus barrier and innate immune defences. Although gastrointestinal colonization can occur under conditions such as antibiotic-induced dysbiosis or severe immunocompromise, oral exposure alone is not considered a typical route for initiating P. *aeruginosa* disease. In the present study, the precise route of infection remains unknown. However, preliminary fluorescence microscopy suggests that bacterial colonization is consistently denser around the eyes during the early stages of infection, in addition to the abdomen (Fig. S2B). While these observations are preliminary and require further investigation, they raise the possibility that infection in our system is initiated through periocular tissues or another external portal of entry, rather than predominantly through ingestion. Consequently, the route of exposure should be carefully considered when interpreting experimental infection models and their relevance to natural or clinical infections.

Interestingly, we also found that specific strains induced different virulence patterns between the two trials within the same host species. These results suggest that phenotypic heterogeneity can also play an important role in modulating virulence. This trial-to-trial variability was most pronounced for strain Lw1047 in the zebrafish assay (Fig. 1d). This pattern raises the possibility that Lw1047 undergoes a more stochastic or switch-like mode of virulence regulation, potentially driven by variable gene expression between exposures, which would be an interesting target for future mechanistic work. While many reasons could explain these findings, differences in gene expression likely explain, at least partly, changes within strains [36, 43, 44]. Additionally, changes in host immunity due to the brood effect or changes in gene expression could also affect the observed virulence phenotype [45, 46], the latter potentially modulated by bacterial gene expression as well [47].

Our study also confirms that *P. aeruginosa* can passively infect a range of organisms, including zebrafish and *C. elegans* [23]. To our knowledge, this is the first evidence that *P. aeruginosa* can incur mortality in zebrafish via passive exposure. While previous studies showed that *P. aeruginosa* PAO1 could induce mortality via microinjection [30], Soest *et al*. [32] reported no mortality in larval zebrafish exposed to *P. aeruginosa* strain PAO1 for 5 h post-fertilization. Here, we show that *P. aeruginosa* strain PAO1 induces significant mortality in 5 days postfertilization larvae. Since our research indicates that bacterial growth does not always correlate with animal mortality, we are confident that mortality is predominantly attributable to the effect of the bacteria rather than other environmental variables such as oxygen depletion or pH changes due to bacterial growth [48]. Because every strain was inoculated at the same concentration and growth stage under identical media conditions, the differences in mortality we observed are attributable to strain identity; whether this reflects genetically encoded virulence factors, strain-specific effects on the local environment (e.g. pH and oxygen), or both remains an open question that will require future studies specifically designed to dissect the mechanistic basis of virulence in this system. Similarly, using microscopy, we systematically screened for the presence of suspected *Paramecium*, a common contamination of laboratory facilities feeding on dead animals [49]. We found that suspected *Paramecium* was only detectable visually 24 h following a mortality incident, suggesting that the parasite likely did not impact our study.

This study also confirms that *C. elegans* is a sound model system for investigating variance in virulence phenotype observed in *P. aeruginosa*. While the virulence of *P. aeruginosa* strain PAO1 was well documented in *C. elegans* [34, 35, 50], our study demonstrates that the nematode is sensitive enough to differentiate virulence patterns between strains. Importantly, our methodology ensured that all nematodes were of the same age and that mortality was thus caused by an active infection by live bacteria accumulating in the lumen of the worm’s intestine as opposed to senescence [33].

Finally, another important consideration in our study is the role of temperature on virulence. According to the protocol for the SK assay detailed by Kirienko and colleagues, we incubated each strain at 37 °C for 24 h and then moved our plates to 25 °C for another 24 h incubation period to allow the bacteria to produce factors necessary for their full virulence in the assay environment [33]. When assessing how *P. aeruginosa* adapts to temperature, researchers found that in PAO1 genes encoding enzymes needed for the biosynthesis of siderophores, pyochelin and pyoverdine were regulated differently at 22 °C in comparison to 37 °C based on their transcriptome analysis [51, 52]. While they found that genes involved in pyoverdine biosynthesis and the pyoverdine operon regulator, ppyR, were upregulated at 22 °C, genes necessary for pyochelin biosynthesis were upregulated at 37 °C [52]. This is important as the siderophore pyoverdine is a key virulence factor that provides *P. aeruginosa* with iron during infection, regulates the production of secreted toxins and disrupts iron and mitochondrial homeostasis in the host [53]. Thus, scientists should further investigate the role of temperature on the virulence of different *P. aeruginosa* strains in other model systems.

The ability to detect differences in larval zebrafish and *C. elegans* mortality between pathogenic strains offers an important model for studying virulence in opportunistic pathogens. We selected the five *P. aeruginosa* strains to represent a broad range of phenotypic diversity, based on isolation site and date. Although the size of our dataset limits our capacity to explore statistical associations between bacterial traits, we can conclude that there is a discernible and repeatable range of virulence responses among the strains.

Using these experimental models opens new avenues for investigating virulence evolution. In particular, because zebrafish develop an adaptive immune system between 2 and 4 weeks post-fertilization [54], infection in larval zebrafish allows for a focused examination of the bacteria’s virulence profile. For instance, a high mortality rate shortly after bacterial exposure may indicate elevated toxin expression or direct tissue colonization [55]. In contrast, a lower but progressive mortality rate could point to virulence factors related to bacterial growth or regulation via gene expression changes or quorum sensing [56, 57]. Further research is required to pinpoint the specific virulence mechanisms of each strain.

Overall, our findings highlight the value of passive exposure in studying the phenotypic diversity of host–parasite interactions across different model systems. This approach offers important insights into pathogen virulence and a path to understanding the development of novel virulence phenotypes in opportunistic pathogens, including the potential emergence of novel pathogens from reservoir species.

## Supplementary material

10.1099/acmi.0.000748.v4 Fig. S1.

## Abbreviations

dpf: days post-fertilization
LB: Luria-Bertani
NGM: nematode growth medium
SK: slow-killing
