# Supplementary material for: Phenotypic heterogeneity in Pseudomonas aeruginosa virulence revealed through passive exposure in zebrafish (Danio rerio) and nematodes (Caenorhabditis elegans)
Source: Access Microbiol. 2026 Oct 20;8(10):000748.v4. doi: 10.1099/acmi.0.000748.v4 (PMC13682699; doi:10.1099/acmi.0.000748.v4)
Supplement: Supplementary file 1 [file acmi-8-00748-s001.pdf]

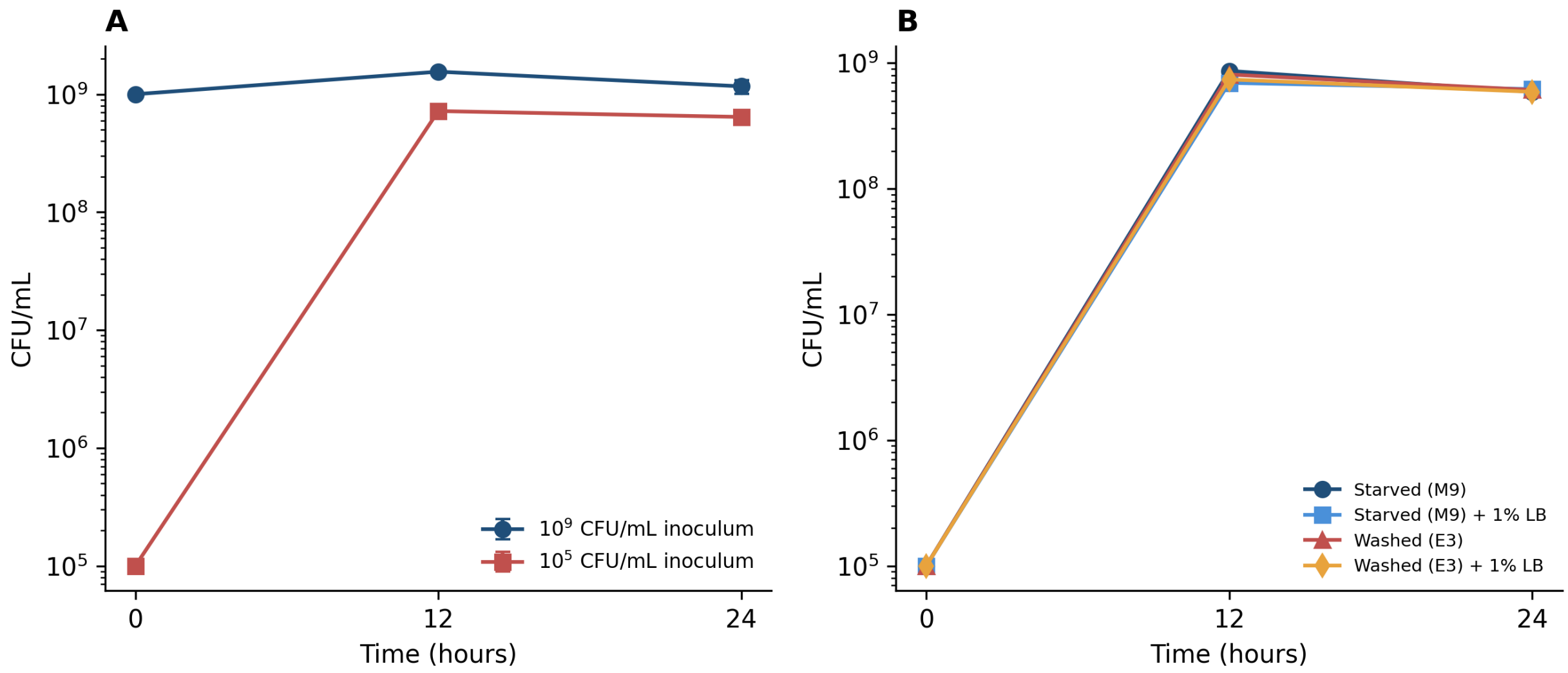


**Supplementary Figure S1.** Growth of *P. aeruginosa* strain PAO1 in 1x E3 medium. (A) Growth over 24 hours following inoculation at $10^5$ or $10^9$ CFU/mL (mean ± SD, n = 3 independent replicates), supporting the selection of $10^5$ CFU/mL as the static immersion inoculum. (B) Growth of bacteria starved in M9 salts for 8 hours or washed in sterile 1x E3 medium, each with or without 1% LB, over 24 hours (single measurement per time point), showing that residual LB nutrients did not drive growth in E3 medium.

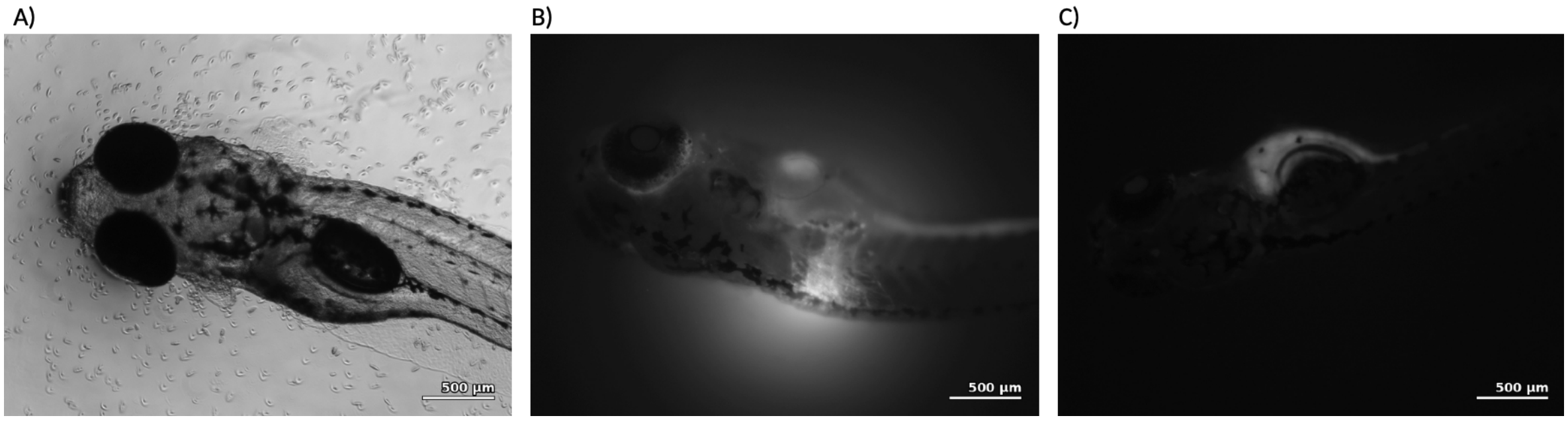


**Supplementary Figure S2.** (**A**) Suspected *Paramecium* (small oval particles, brightfield, 4×) were observed in the E3 medium surrounding a dead control zebrafish larva, consistent with the ciliate bloom described in Methods 2.6; these organisms were not formally identified to species. (**B**) Representative fluorescence micrograph (Nikon Eclipse E600, UV-1A filter cube) of a zebrafish larva exposed to *P. aeruginosa* strain ESP06B, showing a concentration of fluorescence signal around the eye region in addition to the abdomen. This preliminary observation is shown to illustrate, not quantify. (**C**) Representative fluorescence micrograph, acquired under identical imaging settings to (B), of a dead control zebrafish larva, showing minimal background fluorescence by comparison. Scale bars in (A–C), 500 µm; see Methods 2.6 for calibration details.
